# Editorial: The future direction toward immunological issues of allo-and xeno-islet transplantation

**DOI:** 10.3389/fimmu.2024.1487640

**Published:** 2024-09-06

**Authors:** Siyuan Song, Yi Wang

**Affiliations:** ^1^ Department of Neuroscience, Baylor College of Medicine, Houston, TX, United States; ^2^ Center of Critical Care Medicine, Sichuan Provincial People’s Hospital, University of Electronic Science and Technology of China, Chengdu, Sichuan, China; ^3^ Clinical Immunology Translational Medicine Key Laboratory of Sichuan Province, Sichuan Provincial People’s Hospital, Chengdu, China

**Keywords:** allo-islet transplantation, xeno-islet transplantation, immunological challenges, T cell dynamics, mesenchymal stem cells, bioengineering, chronic rejection, pig islet xenotransplantation

The field of transplantation is advancing rapidly, particularly in addressing the immunological challenges associated with both allogeneic and xenogeneic transplants. Recent research highlights key developments in T-cell dynamics, innate immune regulation, and bioengineering innovations. This editorial summarizes these advancements and discusses strategies to overcome the barriers that continue to hinder successful graft outcomes.

## T cell dynamics and molecular mechanisms in islet transplantation

The interaction of T cells within transplanted islets is critical for graft survival. Zhou et al. used single-cell RNA sequencing (scRNA-seq) to explore the molecular mechanisms behind T-cell dynamics in syngeneic and allogeneic islet transplantation. Their findings reveal significant heterogeneity among T-cell subpopulations, including CD4+ T cells, Tregs, and activated CD8+ T cells. The study highlights the differential activation of pathways like interferon-alpha and TNF-alpha signaling, which are crucial for graft outcomes. These insights pave the way for more targeted immunosuppressive therapies tailored to specific immune responses.

## Immunological challenges in allo-beta cell transplantation

Allo Beta Cell Transplantation faces significant immunological challenges, including immune rejection and autoimmunity recurrence. Caldara et al. focus on the interplay between glucose regulation, insulin, and immune activation. The study stresses the importance of standardized immunosuppression protocols, reliable methods for assessing graft rejection, and validated biomarkers for beta cell loss. Emerging strategies include alternative immunosuppressive regimens, targeted autoimmunity prevention, and innovative technologies like CAR-Tregs and genetically modified “stem stealth cells,” which are vital for improving allo-beta cell transplantation outcomes.

## Early microbiological identification in transplantation

The importance of early and accurate pathogen detection in transplant recipients cannot be overstated, particularly in lung transplantation where post-operative infections are a leading cause of morbidity and mortality. Zhang et al. demonstrated that metagenomic next-generation sequencing (mNGS) significantly improves pathogen detection rates compared to traditional microbial culture methods. This early detection capability is crucial for adjusting antimicrobial strategies promptly and effectively, thereby improving patient outcomes. The findings underscore the potential benefits of integrating mNGS into routine clinical practice for transplant recipients to better manage and prevent infections.

## Innovations in bioengineering and stem cell approaches

To address the immunological challenges of islet transplantation, significant advancements have been made in bioengineering and stem cell technologies. Ho et al. discuss innovative strategies such as encapsulation technologies and the development of hypoimmune stem cells. These approaches aim to create an immunoprotective environment around transplanted islets, reducing the need for chronic immunosuppression. The review also explores the potential of human induced pluripotent stem cells (hiPSCs) as a renewable source for islet cells, highlighting the role of gene editing in enhancing their compatibility and function. These bioengineering advancements represent a critical step towards making islet transplantation a more viable and widely applicable treatment for type 1 diabetes (T1D).

## Role of mesenchymal stem cells in enhancing islet transplantation

Mesenchymal stem cells (MSCs) have shown great potential in improving islet transplantation outcomes. Mou et al. highlight the immunomodulatory properties of MSCs, focusing on their ability to reduce immune rejection and support tissue repair. The potential of MSC-derived extracellular vesicles (EVs) to enhance graft survival is also discussed. Despite their promise, challenges like MSC heterogeneity and optimization in therapeutic applications remain. Advanced techniques, including AI and scRNA-seq, are proposed as solutions to these challenges, enabling more personalized treatment strategies.

## Pig islet xenotransplantation: current status and challenges

Pig islet xenotransplantation offers a promising alternative to human donor pancreases, addressing the growing demand for islet transplants. Cooper et al. review the progress in this field, focusing on the development of gene-edited pigs that are more compatible with human recipients. The transplantation of neonatal pig islets (NICC) shows several advantages, including lower costs and simpler isolation processes. However, challenges such as the instant blood-mediated inflammatory reaction (IBMIR) and the need for effective immunosuppressive therapy persist. The review concludes that with continued advancements, pig islet xenotransplantation holds significant potential for clinical application.

## Chronic rejection in lung transplantation: implications for islet transplantation

Chronic rejection remains a significant obstacle in lung transplantation and serves as a relevant model for understanding similar challenges in islet transplantation. Heigl et al. investigate the nature of chronic rejection in a murine orthotopic lung transplant model, revealing that rejection may begin as an arterial response rather than being airway-centered. These findings challenge traditional understandings and suggest that a broader perspective, including vascular and pleural involvement, is necessary for improving graft outcomes. This research has important implications for islet transplantation, where chronic rejection remains a critical challenge.

## Advancements in innate immune regulation

The innate immune response, particularly by macrophages, poses a significant barrier to graft survival in islet transplantation. Duan et al. review strategies to regulate this response, including drug therapies, optimization of islet preparation, and cotransplantation with MSCs. The study highlights the potential of blocking Toll-like receptor 4 (TLR4) signaling and inhibiting the NLRP3 inflammasome to reduce macrophage-induced inflammation, which is crucial for improving islet graft survival.

## Optimal conditions for islet culture in xenotransplantation

Maintaining the viability and functionality of porcine islets during long-term culture is essential for successful xenotransplantation. Sakata et al. investigate the effects of different temperatures on the culture of adult porcine islets, concluding that 37°C is optimal for preserving islet morphology, promoting cell proliferation, and restoring endocrine function. This research provides valuable insights into the culture conditions necessary for maintaining the quality of porcine islets, which are a promising source for xenotransplantation.

Advancements in understanding T-cell dynamics, immunomodulatory therapies, and bioengineering are paving the way for improved outcomes in both allo- and xeno-islet transplantation. These developments not only enhance graft survival but also address broader immunological challenges in transplantation. Continued integration of these strategies will be key to overcoming barriers and advancing the field.

